# Ten simple rules for international short-term research stays

**DOI:** 10.1371/journal.pcbi.1005832

**Published:** 2017-12-07

**Authors:** Diego A. Forero, Sandra Lopez-Leon, George P. Patrinos

**Affiliations:** 1 Laboratory of NeuroPsychiatric Genetics, Biomedical Sciences Research Group, School of Medicine, Universidad Antonio Nariño, Bogotá, Colombia; 2 PhD Program in Health Sciences, School of Medicine, Universidad Antonio Nariño, Bogotá, Colombia; 3 Global Drug Development, Novartis Pharmaceuticals Corporation, East Hanover, New Jersey, United States of America; 4 Department of Pharmacy, University of Patras School of Health Sciences, Patras, Greece; 5 Department of Pathology, College of Medicine and Health Sciences, United Arab Emirates University, Al-Ain, United Arab Emirates; Dassault Systemes BIOVIA, UNITED STATES

## Introduction

Because science is a global endeavor, international mobility is common among researchers and academics around the world [1,2]. Short-term research stays (from a few weeks to a few months), which do not involve a change of employer or affiliation, are the main form of international mobility [3]. This type of “brain circulation” [4] increases collaborations, creates networks, improves career prospects, facilitates the generation of high-impact publications, gives access to international funding, and nourishes ideas through exposure to different methods and scientific skills [5–7]. In this article, we present 10 simple rules for better and more productive experiences of international scientific mobility, which might be helpful for scientists in all stages of their careers, particularly for MSc and PhD students [8].

## Rule 1: Select your host institution

The affinity in academic and research topics between the host and the guest laboratories is a priority [9,10]. Other affinities (e.g., nationality, language, or culture) come second. However, do take into account that countries with little mobility embrace diversity less easily [11]. Studies have shown that the main drivers of choosing a host institution are having an outstanding faculty or research team; the infrastructure and facilities; and the expertise, excellence, prestige, or high quality of the foreign institution in a certain area [11–13]. There are several ways of finding an adequate host. Simple ways are to send emails to authors who publish work in your field of interest, to ask supervisors and your network, or to attend short courses and conferences. In addition, you can search websites of universities, academic centers, regulatory agencies, and industry. Some scientific societies have a section in which possibilities for short stays are announced, and an industry posts their intern research positions in traditional job posting websites (e.g., indeed.com, linkedin.com, monster.com, experteer.com). Contacting the international office of the host university would help with some administrative processes and for exploring additional options. In many cases, the administrative staff of the host department is quite helpful in supporting logistical aspects of the international visit.

## Rule 2: Plan ahead carefully

Planning for a research stay abroad takes time in order to organize in advance the different aspects; namely, adequate time in the host institution, plane tickets, accommodation, visas, insurance and permissions, details of the experiments to be carried out, and rotations in other laboratories and/or centers, among others [9,14]. It is imperative that the necessary paperwork for the exchange student and/or staff be executed well in advance. Scientists should not leave their country before these documents are signed by both parties. All details (time, remuneration in certain cases, casualty and legal liability insurance, among others) should be laid out in the contract. This requires adequate coordination between the members of the host and guest laboratories and institutions. It is key to have a clear understanding of the timelines of the main regulatory processes required in the host institution, such as approval by the local research ethics committee.

Once you identify the host country, it is essential that you visit the website of the embassy and/or consulate in your location and that you contact the international office of your institution to find out the requirements and the time it takes to issue the required travel documents. In addition, the embassy should be able to provide extra information, such as sources for funding, and advise you if any (extra) vaccines are needed. Make sure your health insurance covers you in the host country; otherwise, get international insurance. If you are planning on driving, and your license is not valid in the host country, obtain an international driving license (usually valid for up to one year).

## Rule 3: Define funding needs and sources for your research stay in advance

Obtaining funding for mobility is the biggest challenge for researchers [3]. Costs of reagents or the use of specialized equipment requires funding to cover those expenses, which might be charged to the host or the guest’s laboratory. Sometimes, this is covered by overheads available in host institutions as part of their research grants. In several cases, the results from a research stay become pilot data for future collaborative projects aimed at obtaining more funding [9]. If neither your institution nor the host institution provides funding, you can search for grants given by other institutions, include the international stay in your grant proposals, or consider saving and paying for your stay yourself. A survey of almost 9,000 PhD students found that more than 20% used personal savings to finance stays abroad [15].

## Rule 4: Respect the organization of the host institution

There are differences between institutions in the way that departments are organized, and some of these differences might be amplified because of dissimilarities between countries (and more importantly, cultures). Respect for the organization of the host institution might facilitate an adequate development of the activities to be carried out by the guest scientist. Knowing details of the organization, structure, and dynamics of the host institution helps in the process of integrating the guest researcher. Before you travel, you can talk to several people working in the department and to past visiting researchers to understand what to expect.

During your stay, follow the wisdom of the saying “When in Rome, do as the Romans do.” Do not take things for granted, and when in doubt, ask. Never share data from the organization with outside people and do not use materials and/or programs or analyze data without prior written authorization.

## Rule 5: Be prepared for integration into the host laboratory

It is key that you look for a potential direct mentor or adviser (in several cases, it is a scientist different from the principal investigator [PI] of the host laboratory) who takes a key role in your constant supervision and academic support from an early moment. You are encouraged to attend laboratory meetings in advance of your stay—e.g., using videoconferencing tools—to have a better understanding of the dynamics of the host laboratory. You need to have a good understanding of the language of the country of the host laboratory to facilitate adequate communication with the other members. In some cases, English is the main option, but in several countries, other languages are needed. Integration into the host laboratory would facilitate the completion of key goals defined in advance of the research stay and an adequate reporting of findings and advances (e.g., presentations in laboratory and department meetings and preparation of manuscripts, among others).

Expanding your scientific knowledge is the priority. You should not see the international experience as merely a means to boost your resume or as a paid leisure trip. Leisure should be seen as secondary, and extra time should be set aside for it either by leaving some hours at the end of the workday or by adding extra days to your stay.

A key point for a successful research stay is that the scientific activities to be carried out at the host institution remain the main priority [10,11,14]. This means that there is a need (particularly for students) for an adequate articulation to the guest’s home laboratory of the activities to be carried out at the host institution. Take into account that part of the collaborative work can be finalized in your own country, so focus on the activities for which one needs to be physically present face-to-face—such as scientific discussions and the use of databases, computer programs, laboratory samples, materials, and instruments that can only be used at the host institution.

## Rule 6: Define authorship and acknowledgments in advance for the products of your research stay

As with other type of collaborative endeavors, it is fundamental to define in advance the authorship of the possible publications and/or conferences resulting from the research stay. If authorship and conference presenters are not clarified beforehand, this usually leads to unpleasant situations between research groups and may lead to discontinuation of collaborations [9]. Take into account that guidelines for assigning authorship vary between fields; therefore, make sure to understand what to expect. Find out what the requirements are to be considered an author. Define who is going to be first author, how the order of the author list will be determined, and who is going to be the corresponding author. In some areas, being the last author means that you were the PI, while in others it means that you contributed the least. Although listing authors in order of involvement seems straightforward, a study showed that more than two-thirds of 919 corresponding authors disagreed with their coauthor order [16].

In addition to defining the authorship of publications and/or conference proceedings in advance, other types of acknowledgment of the collaborations are important [11], such as presentations in scientific events and in other types of documents (MSc and PhD thesis, final reports of projects, future grant applications, among others) [17].

## Rule 7: Learn from the differences

In several cases, one of the most valuable experiences from international mobility is learning different styles of doing science [18]. It is also a good opportunity to practice other languages and to have an immersion in other cultures. Going to lectures or laboratory meetings and journal clubs is a great way to learn from the academic environment of the host institution.

Go with the mentality that you are the one who has to adapt to their culture and way of working; do not expect people to adapt to you. A good resource to understand the differences between your culture and the culture you are visiting is the book *The Culture Map* [19] and the series of interviews that *Science* published for international scientists (http://www.sciencemag.org/careers/2011/08/international-mobility). If the language and culture are completely different, then there may be benefits of having a cultural coach who can explain unfamiliar hierarchies, conflict management, and body language.

## Rule 8: Try to resolve problems in an adequate way during your research stay

Inconveniences between research groups and/or members are frequent, and these conflicts might be of particular relevance in research stays of international guests [10,11]. An early identification of possible inconveniences is key in order to manage them in a transparent and cordial manner [9]. When dealing with other cultures, communication is essential because many of the problems arise because of misunderstandings. If something is bothering you, or if you expect something, say it. Let them know that you are open to their feedback and that you would like to know if there is something that was expected from you. Keep in mind that things that are obvious in your country may not be so in your host country; therefore, there is a need for you to be flexible and open-minded and to try to adapt as much as possible.

## Rule 9: Explore other options for carrying out collaborations

The use of videoconferencing, file sharing services [20], and webinars facilitates some facets of international collaborations because these resources are less expensive and complex than international flights [21]. These strategies, which might save costs and personnel time and effort, might be of particular importance for researchers from resource-limited countries [22]. Consider collaborations with other sectors, including industry, contract research organizations, regulatory agencies, and other universities. Diverse teams that are able to complement each other often lead to the development of innovation and creativity [23].

## Rule 10: You can be a host as well

Invite a researcher to your institution. Having an international researcher in your institution can bring the same advantages to the host and to the visitor, including collaborations, networks, and exposure to different methods and scientific skills; plus, visiting guests could give lectures and bring experience and new ideas into your laboratory or institution. Involve your supervisors, legal, and human resources in the idea from an early stage because they will be essential in helping with the paperwork and accommodations. When you write a research grant, you can consider including an international visitor.

## Conclusion

Please note that every single rule and all advice given in this article can also be applied by the host researcher. Several of the rules are related to general aspects of scientific collaborations, but they have particular characteristics inherent to the dynamics of short-term international scientific mobility. The advancement of international communication and geographical mobility has made “brain circulation” accessible. However, when visiting research institutions abroad, there are many details that need to be taken into account that are easily missed. Therefore, these rules will be an essential source of guidance when planning your visit. Congratulations on your next international short stay; you will have one of the best experiences in your academic life.
